# Exploring the influence of sterilisation and storage on some physicochemical properties of coconut *(Cocos nucifera L.) *water

**DOI:** 10.1186/1756-0500-4-451

**Published:** 2011-10-27

**Authors:** Adolf K Awua, Edna D Doe, Rebecca Agyare

**Affiliations:** 1 Cellular and Clinical Research Centre, Radiological and Medical Sciences Research Institute, Ghana Atomic Energy Commission, Accra, Ghana

## Abstract

**Background:**

Fresh coconut (*Cocos nucifera L*) water is a clear, sterile, colourless, slightly acidic and naturally flavoured drink, mostly consumed in tropical areas. It is a rich source of nutrients and has been used for medical purposes. This study was designed to investigate changes in selected characteristics of coconut water after autoclaving, gamma irradiation and storage. Also, the study was designed for assessing the possibility of measuring the growth of bacterial in fresh, stored or sterilised coconut water using turbidity measurements (at wavelengths between 600 nm and 800 nm) or by dry biomass determinations.

**Results:**

Portions of coconut water aseptically extracted from the matured fruit, (average pH of 6.33 ± 0.17) were either stored at 4°C, autoclaved at 121°C for 20 min., or irradiated with gamma rays at 5 kGy. Subsequent changes in selected characteristics were determined. Autoclaving, gamma irradiation and long term storage of coconut water at 4°C resulted both in the development of a pale to intense yellow colour and changes in turbidity. After storage, the dry matter content of fresh, autoclaved and irradiated coconut water by 52.0%, 23.5% and 5.0% respectively. There were also significant differences in the UV spectra before and after sterilisation and during the storage of the coconut water. Although changes in total carbohydrates were observed, they were not significant (*p > 0.05*).

**Conclusions:**

The enormous differences in the characteristics before and after storage suggests that the use of turbidity and dry biomass measurements for measuring the growth of bacteria in fresh, autoclaved and gamma irradiated coconut water before storage is practicable without any possibility of interference by the innate turbidity, colour and dry matter of the coconut water. However, this is not practicable after storing the coconut waters at 4°C, since there were increases in the turbidity and dry matter of the coconut water to levels that will mask the turbidity of a growing bacteria culture.

## Background

Coconut (*Cocos nucifera L*) water, also referred to as coconut juice, is a refreshing natural drink common and mostly consumed in the tropical regions of the world [1,2]. It is a clear, colourless, sweet, naturally flavoured slightly acidic drink, with reported pH ranging between 4.2 and 6.0 [3,4].

Over six decades of research has shown that coconut water contains proteins, fats, and is rich in carbohydrates and nutritionally important elements (potassium being the most abundant) [3-7]. It is also a rich source of essential amino acids (lysine, histidine, tyrosine and tryptophan), fatty acids, glucose, fructose, cellulose, sucrose, and organic acids such as tartaric, citric and malic acids [7-10].

Coconut water's rich enzyme systems include very effective and selective reductase [1], polyphenol oxidase (PPO) and peroxidase (POD). These are involved in its development of a brownish colour when it is exposed to air for a long time [11]. Based on its content and properties, coconut water has been used in the treatment of child and adult diarrhoea, and gastroenteritis as well as for urinary stone dissolution, short-term intravenous hydration and protecting against gastrointestinal tract infections [12].

While still in an undamaged fruit, coconut water remains sterile and stable, but it may become unstable when extracted from the fruit and stored for a few days at 4°C. In Ghana, it is commonly available in the coastal areas and mostly consumed fresh and directly from the fruit. However, the conduct of its vendors and consumers exposes it to a high risk of contamination with bacteria, specifically coliforms.

Assessing the risk of bacteria infections through the consumption of coconut water is made a difficulty since there are limited reports that show the survival and growth of pathogenic bacteria in coconut water. Considering the risk of bacteria contamination of coconut water in Ghana, the possibilities of survival and growth of bacteria in coconut water and the potential for the use of coconut water as a bacterial growth media in resource-limited countries/laboratory like ours, we initiated this study. We studied some characteristics of coconut water obtained from locally grown crop. We also investigated how these change with autoclaving, gamma irradiation and storage. We report here, changes in colour, turbidity, dry matter, pH and UV absorbance as well as the influence of temperature on the visible light absorbance of coconut (*Cocos nucifera *L) water after autoclaving, gamma irradiation and storage at 4°C. An assessment of the possibility of measuring bacterial growth by measuring the turbidity and dry biomass of bacteria cultures in fresh, stored or sterilised coconut water was also presented.

## Method

### Extraction of Coconut Water

Three matured green coconut fruits, with no visible damage, were obtained from the local market (not more than 48 hour after harvest). Parts of the mesocarp and endocarp were removed to expose the endosperm, without damage. After surface sterilisation with 70% ethanol and exposure to UV light (240 nm) in a Biosafety Class II Cabinet (Clean Air Systems Chennai, India), the endosperm was cut-out with a sterile surgical blade and the water transferred to a sterile Ducan bottle. After estimating the volumes, portions were stored at 4°C and -20°C. The pHs of the fresh coconut water, the coconut water after two weeks and one month of storage at 4°C were determined (HANNA pH 211, Microprocessor pH meter, Sigma-Aldrich, St. Louis USA). The sterility of each coconut water extract and stored coconut water was tested by inoculating nutrient agar plates and 10 mL Luria Bertani broth (Sigma-Aldrich, St. Louis USA) with 150 *μl *of each and incubating at 37°C for 24 hours.

### Sterilisation of Coconut Water

About 200 mL of fresh coconut water was autoclaved at 121°C and 1.5 bars for 20 minutes (NAPCO model 9000-D, Pegasus Sci. Inc., Rockville USA). Another 250 mL was radiated with 5 kGy of gamma radiation (with a dose rate of 10 kGy/h at 10 ± 0.5°C. Their pHs were determined and portions stored at -20°C until further use and at 4°C for further study.

During storage, the samples were intermittently transferred to room temperature (about 25°C), for not more than 30 minutes in a day, once every week for one month, to simulate the normal situation that may apply in the use of stored liquid bacteria media.

### Absorbance Spectra

The absorbance of the fresh, autoclaved and radiated coconut water within a wavelength range of 200 nm and 600 nm were measured (UV-VIS 1210 Spectrophotometer, Shimadzu Corp., Columbia MD, USA), with repeats after two weeks and one month of storage at 4°C. All determinations were performed in duplicates. Temperature related absorbance spectra for stored fresh coconut water were obtained by measuring it absorbance (between 300 nm and 540 nm) after it has been maintained at a temperature (ranging from 35°C to 99.8°C) for 10 minutes.

### Dry Matter Content

The amounts of dry matter in fresh, autoclaved and radiated coconut water were determined before and after one month of storage at 4°C. An aliquot of 1 mL of each was centrifuged at 5000 rpm (Eppendorf centrifuge 5415 C) for 15 minutes. The pellets were air dried at 60°C (GFL -7601 Hybridisation Incubator, GFL mbH, Burgwedel, Germany) and weighed repeatedly until a constant weight was obtained (Sartorius CP64, Data Weighing Systems, Grove IL, USA). All determinations were performed in triplicates.

### Total Carbohydrate

Total carbohydrate was determined according to the Anthron's method as reported by Eklöf *et al*., [13].

### Statistical Analysis

MS. Excel microcomputer software (Microsoft Corporation) was used to obtain descriptive statistics (averages, standard error etc) and percentage changes (increases and decreases) in measured parameters. The student t-test was used to analyse for statistical significance in the differences in dry matter and the linear regression model was used to analyse the relationship between abhorrence and temperature.

## Results

### Coconut Water and Related Changes

The absence of colonies on nutrient agar plate and the lack of change in the turbidity of LB broth inoculated with coconut water indicate that the extraction process was aseptically performed and the coconut water was sterile. The average volume of water obtained ranged between 250.0 and 300.0 mL (average of 266.7 ± 16.7 mL) with an average pH of 6.33 ± 0.17. The thickness of the endosperm ranged between 0.5 cm and 0.6 cm (average of 0.53 ± 0.03 cm).

**Figure 1:**
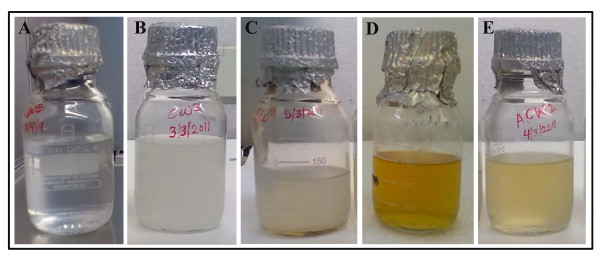
**Pictures of coconut water before and after sterilisation and storage at 4°C**. **(A)**. Fresh coconut water after extraction. **(B)**. Fresh coconut water after two weeks of storage at 4°C. **(C)**. Irradiated coconut water after four weeks of storage at 4°C (no significant difference to the observation before and after two weeks of storage). **(D)**. Autoclaved mixture of coconut water before storage. **(E)**. Autoclaved coconut water after two weeks of storage. For B, C and E, the increase in turbidity/cloudiness of the water masked the intensity of the yellow colour; however, it was observable after centrifugation.

**Figure 2:**
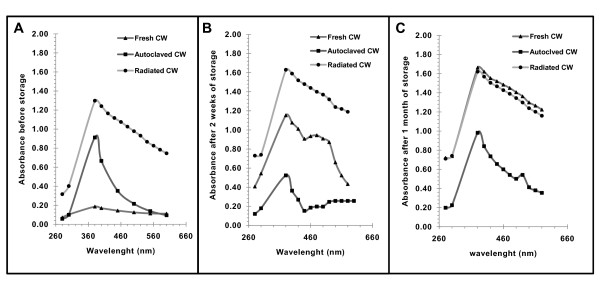
**Visible-light spectra of coconut water before and after sterilisation and storage at 4°C**. **(A)**. After autoclaving at 121°C and 15 psi for 20 minutes and gamma irradiation at 5 kGy, absorbance of fresh, autoclaved and irradiated coconut water within the visible light region were measured. **(B)**. After two weeks of storing the fresh, autoclaved and gamma irradiated coconut water at 4°C, their absorbance were measured at 20 nm interval within the visible light region. **(C)**. After one month in storage at 4°C the absorbance measurements were repeated as in B, These were all plots of the average of three absorbance readings at each wavelength. Absorbance readings at wavelengths from 320 nm to 340 nm were very unstable and as such were excluded from the plot.

After the gamma irradiation (at 5 kGy) and autoclaving, a pale and intense yellow colour was observed respectively (Figure 1). However, gamma irradiated, autoclaved and fresh coconut water both recorded a wavelength of maximum absorbance (λ_max_) of 385 nm (Figure 2A). Relative to the fresh coconut water (FCW), a stable and an increase in turbidity was observed for the autoclaved coconut water (ACW) and irradiated coconut water (RCW) respectively. These differences in turbidity were confirmed by the visible spectrum (Figure 2A) at wavelengths between 580 nm and 600 nm; where the absorbance of the autoclaved coconut water was just below that of the fresh coconut water, and both were lower than that of the irradiated coconut water. I.e. ACW = FCW < RCW.

The visible light spectra of all three samples of coconut water (fresh, autoclaved and irradiated) before storage was presented as Figure 2A. After two weeks of storage, fresh coconut water developed a pale yellow colour, increased in turbidity (Figure 1) and opened with a puff sound. Its average increase in absorbance across the range of wavelengths (Figure 2B) was also extensive, (by about 500%). However, a slight increase in turbidity with no change in the intensity of the pale yellow colour and a mild increase in average absorbance (about 25%) were observed for irradiated coconut water (Figure 2B).

On the other hand, there were changes in the turbidity and the intensity of the yellow colour of autoclaved coconut water (Figure 1D and 1E). The related spectra indicated an increase in absorbance by about 108% between 560 nm to 600 nm and a decrease by 41% between 280 nm and 500 nm respectively (Figure 2B).

After four weeks of storage, the absorbance of the autoclaved coconut water was restored to almost what it was before storage, while the absorbance of the irradiated coconut water did not show any appraisable change. On the other hand, the absorbance of fresh coconut water further increased by about 44% and became intense yellow (Figure 2B and 2C). However, the increase in the intensity of the yellow colour was masked by the increase in turbidity; the true colour was observed after centrifugation to pellet the suspended matter.

In summary, the autoclaved coconut water remained relatively a clear medium while the fresh and irradiated coconut water increased in turbidity during storage. For the fresh and irradiated coconut water, sediments were observed at the base of the containing bottles by the 4^th ^week of storage. It was also observed that the cloudiness/turbidity of various stored coconut water samples masked their yellow colour. It should be noted that absorbance between 600 nm and 700 nm has been used extensively for measuring the growth (increase in cells) of bacteria in liquid cultures; as such a cloudy solution/medium will most likely cover-up little changes in turbidity of a growing bacteria culture, particularly at its lag phase.

### Changes in Dry Matter

**Figure 3:**
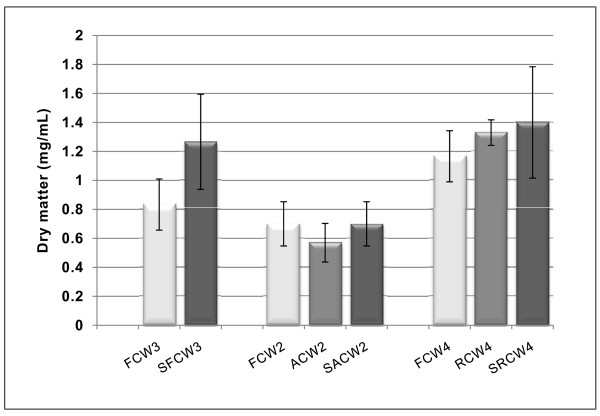
**Dry matter content of coconut water before and after sterilisations and storage at 4°C**. Aliquots (1 mL) of the different samples of coconut water were centrifuged and the supernatant discarded (this was done in triplicates). The pellets were dried at 60°C and weighed every 30 minutes until a constant weight was obtained (Sartorius CP64 weighing balance). The averages of three weights for each sample were plotted with the standard error indicated as error bars. The large standard errors (error bars) observed for the irradiated and fresh coconut water after storage were as a result of the fact that the sediments settles to the base of the bottle very fast when mixed before measuring out the needed volume for analysis. FCW2, FCW3, FCW4, = are different samples of fresh coconut water; SFCW3 = stored fresh coconut water sample; ACW2 = autoclaved coconut water sample; SACW2 = stored autoclaved coconut water sample; RCW = irradiated coconut water; SRCW4 = stored irradiated coconut water sample.

The changes in turbidity observed were further studied by determining the dry matter (mg/mL) of the coconut water samples before and after the one month storage period (Figure 3 and table 1). The dry matter content of three different samples of fresh coconut water did not differ significantly (*p > 0.05*) with each other. After autoclaving, the dry matter reduced by 19.1% (not significant, *p > 0.05*) but increased by 14.3% after gamma irradiation (not significant, *p > 0.05*). After four weeks of storage at 4°C, fresh, autoclaved and irradiated coconut water, increased in dry matter by 52.0%, 23.5% and 5.0% respectively. Regarding the combined effect of autoclaving and storage, and irradiation and storage, it was observed that the latter resulted in a 20% net increase in dry matter while the former resulted in no net change in dry matter within the period of storage.

**Table 1:** Changes in dry matter content of coconut water due to sterilisations and storage

Samples	% Change in dry matter		
	
	After treatment	Stored	Combination
Fresh coconut water	--	52.0	--
Autoclaved coconut water	-19.1	23.5	0.0
Radiated coconut water	14.3	5.0	20.0

The coconut water was autoclaved at 121°C and 15psi for 20 minutes or Irradiated at 5 kGy. All storage was at 4°C for 1 month.

### Changes in UV Spectra

**Figure 4:**
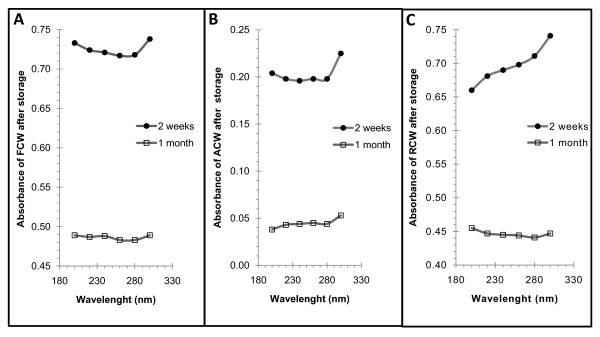
**UV spectra of fresh, autoclaved and irradiated coconut water after storage for two weeks and one month**. After two weeks and one month of storage of; **(A)**. Fresh coconut water, **(B)**. Autoclaved coconut water (121°C and 15 psi for 20 minutes) and **(C)**. Gamma irradiation (5 kGy) coconut water the UV absorbance between 200 nm and 300 nm were measured at an interval of 20 nm in triplicates. These plots are of the average of three absorbance readings at each wavelength. FCW = Fresh coconut water. ACW = Autoclaved coconut water. RCW = Irradiated coconut water.

In comparison to the UV spectrum of fresh coconut water stored for two weeks, a dramatic reduction in absorbance (69.5% to 72.0%) was observed across all wavelengths for the spectrum of autoclaved coconut water stored for the same period. However, the pattern of both spectra was not so different (Figure 4A and 4B). With respect to gamma irradiated coconut water stored for the same period, UV absorbance at wavelengths less than 260 nm were extensively reduced while those at wavelengths greater than 260 nm changed marginally, resulting in a drastic change in the pattern of the spectra (Figure 4A and 4C).

After one month of storage, the overall UV absorbance of fresh coconut water reduced by an average of 30%, resulting in a slight change in the pattern of the spectrum (Figure 4A). For autoclaved coconut water, large reductions (between 80% and 76%) in the overall absorbance were observed but with a resultant marginal change in the pattern of the UV-spectrum (Figure 4B). With regards to irradiated coconut water, stored for the same period, a reduction (between 30% and 40%) and a significant change in the pattern of the UV spectrum was observed (Figure 4C).

### Changes in pH

An average pH of 6.33 ± 0.17 was recorded for the fresh coconut water, however, a reduction by 1.5 units was observed after autoclaving and gamma irradiation (table 2). After one month of storage, the pH of the fresh coconut water reduced by an average 2.3 pH units while those of autoclaved and irradiated coconut water, did not change. A mixture of samples of fresh coconut water studied also recorded similar observations.

**Table 2:** pH variations in coconut water before and after sterilisation and storage

Sample	Initial pH	Magnitude of pH decrease after		
		
		Autoclaving	Irradiation	Storage
Fresh coconut water	6.0-6.5	1.5	1.5	2.0
Mix coconut water	6.5	1.5	--	2.5
Autoclaved mix coconut water	5.0	--	--	0.0
Autoclaved coconut water	5.0	--	--	0.0
Radiated coconut water	4.5	--	--	0.0

The coconut water was autoclaved at 121°C and 15psi for 20 minutes or Irradiated at 5 kGy. All storage was at 4°C for 1 month.

### Temperature and Colour Change

**Figure 5:**
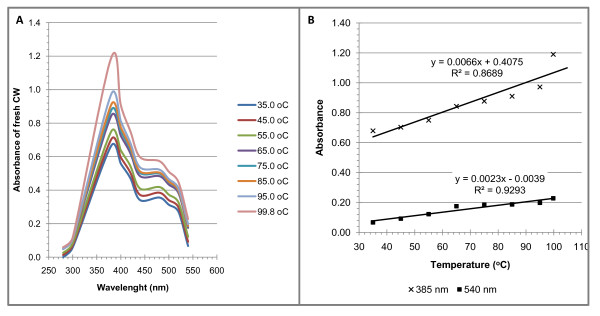
**Visible light spectrum and temperature profile of fresh coconut water stored for two weeks**. Duplicates of 10 mL of fresh coconut water were incubated at 35°C with an increase in temperature by 10°C every 10 minutes. At the end of each incubation period, absorbances were measured at wavelengths intervals of 20 nm starting from 280 nm to 540 nm, **(A)**. presents the visible light spectrum at each incubation temperature. **(B)**. presents the regression of the absorbance at the wavelength of maximum absorbance (λ_max_, 385 nm) and temperature as well as that between the absorbance at 540 nm and temperature.

There was an increase in the absorbance of stored fresh coconut water, (at 4°C for two weeks) as its temperature increased between 35°C and 99.8°C (Figure 5A and 5B) with a λ_max _at 385 nm. An observable larger increase in the intensity of the yellow colour was seen at temperatures 95°C or higher.

### Changes in Total Carbohydrate

**Figure 6:**
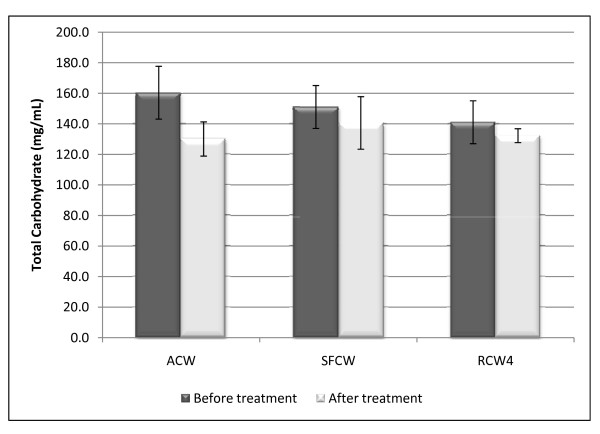
**Total carbohydrate content of fresh and sterilised coconut water**. The total carbohydrate was determined by the Anthrone's method. The plots are of triplicate determination with the standard error indicated as the error bars. SFCW, = Stored fresh coconut water; ACW = autoclaved coconut water; RCW = irradiated coconut water. 'Before treatment' and 'After treatment' imply before and after, autoclaving for ACW, storage for SFWC and radiation for RCW.

Although autoclaving, irradiation and storage resulted in the reduction of the total carbohydrate content of coconut water by about 18.9%, 6.2% and 6.9% respectively (Figure 6), the influence of the two sterilisation methods on the total carbohydrate content of coconut water was not significant (*p > 0.05*).

## Discussion

The observed sterility of the extracted, sterilised and stored coconut water imply the changes observed for the fresh, stored and sterilised coconut water were not due to contaminating microbes as was the case in the study by Chowdhury *et al*., [14] who also studied coconut fruits obtained from the local market, with no data on the age of maturity.

Although the maturity of the fruit could not have been determined accurately, the thickness of the endosperm were within the range (between 4 and 10 mm) reported for 12 month old matured coconut fruit by Santoso, *et al*., [8]. The volumes of coconut water (266.7 ± 16.7 mL) were lower than those reported (350 mL to 500 mL) by Campbell-Falck *et al*., [7] and Santoso, *et al*., [8]. These variation in volume of coconut water, as well as variations in enzyme activity, pH and nutritive values have been reported to be due to differences in plant variety, locality of cultivation and the age of maturity (before and after eighth month), [15,16].

The observed differences in colour and turbidity (Figure 1) were confirmed in the visible spectra (Figures 2A-C). When exposed to oxygen, polyphenol oxidases catalyse the hydroxylation of monophenols, such as in tyrosine (present in coconut water), to *O*-diphenols; these are further oxidised to *O*-quinones and semiquinone, which polymerised to different extents, leading to the yellow, orange or brown colours [11,16-18]. The added heating to 60°C or above enhances the activity of the enzymes (PPO and POD), which is reduced/lost at temperatures above 90°C [11]. Such colour changes (due to PPO and POD) have been reported for Mueller-Hinton broth after autoclaving [19].

The change in colour after gamma irradiation is explainable on similar bases, since irradiation with gamma rays (ionizing radiations) leads to the formation of hydroxyl phenal radicals (phenol side group of tyrosine and gallic acid) [20] which then serves as the precursor for the coloured *O*-quinones.

The visible light spectra after two weeks of storage (Figure 2B) also indicate changes in colour and turbidity of fresh, autoclaved and irradiated coconut water. Similar observations were made by Puchakawimol *et al*., [18] where fresh coconut water became orange-yellow in colour and increased in the saturation of colour on the later days of storage at room temperature. Also, Chowdhury *et al*., [14] showed that after one month of storage at 0°C, fresh coconut water changed in colour and flavour, increased in pH and turbidity with the formation of gas in addition to being contaminated with fungi.

These observations suggest that the reactions of polyphenol oxidase and peroxidase, and the formation of the insoluble matter continued during storage at 4°C. The decrease and subsequent increase in colour intensity of autoclaved coconut water after 2 weeks and one month respectively, suggests that polyphenol oxidase and peroxidase (which catalyse reversibly, both oxidation and reduction reactions [11,18,16,21]) in response to the reduction and increase in oxygen (due to storage in an air tight container for two weeks, and after opening the air tight container respectively) converted the chromophore and its precursors reversibly. However, the continuous increase in the turbidity and absorbance above 540 nm (Figure 2B and 5), suggests that the reactions forming the insoluble matter and colour were still occurring and that the enzymes involved where also heat resistant.

The increase in colour and turbidity of irradiated coconut water for two weeks with no significant increase after an additional two weeks needs further investigation for a clearer understanding, since it is generally accepted that the activity of POD and PPO are not significantly affected by low dose (about 5 kGy) gamma radiations [22]. However, Frylinck *et al*., [23] detected a partial inactivation of these enzymes in mango fruit after low dose gamma irradiation. Therefore, we ask, is it that in this study of coconut water only small quantities of phenal radicals were produced after irradiation? Or is it that the activities of these enzymes were greatly reduced or lost after irradiation? A study that will measure the activity of these enzymes and estimate the changes in the concentration of radical in fresh and irradiated coconut water before and at regular time intervals during storage may help to throw more light on this.

The results of the dry matter (Figure 3 and table 1) correlated with the changes in the absorption spectra between the wavelength 560 nm and 600 nm (Figure 2A-C), as an indication of turbidity. Autoclaving resulted in a decrease in dry matter by 1.1%, seen as a lower absorbance of autoclaved coconut water compared to that of fresh coconut water (Figure 2A). Conversely, irradiation resulted in an increase in dry matter (Figure 2A) and an associated increase in absorbance in that same range. Similar correlations were observed after storage. (Figure 2B and 2C; table 1).

The results presented as Figure 4 demonstrate the changes in UV absorption of coconut water due to a combination of sterilisation and storage. Since coconut water is a mixture and/or a solution of various biomolecules, including aromatic amino acids (tyrosine), gallic acids, flavour giving aromatic hydrocarbons, biphenal products of the enzymes POD and PPO and others as reported by Yong *et al*., 2009 [24], the significance of the changes in UV absorption in relation to its biochemical and nutritional components cannot be evaluated with this data. However, these changes provide evidence for the occurrence of both physical and biochemical reactions (to different extents) after sterilisation and during storage at 4°C. The interactions between biomolecules have been shown as one of the physical processes by which the UV spectra of a molecule may be changed [25]. On the other hand, the reformation of aromatic system via proton and electron rearrangement or perturbations as a result of exposure to radiations, are some of the chemical process that influence and change absorption in the near UV region [26].

The reduction in pH, as was observed after autoclaving, irradiation and storage (table 2) was a result of the formation of the phenol radicals and oxides, leading to the formation of the yellow colour. Stored fresh coconut water experienced the greatest change in pH probably because its POD and PPO activities were not reduced (as was expected after autoclaving and gamma irradiation) and therefore contributed to a higher release of protons.

The temperature profile (Figure 5A and 5B) indicates that the changes in absorbance at 385 nm (indicative of colour) and 540 nm were linearly related to an increase in the temperature of stored coconut water (*R^2 ^of 0.87 and 0.93 *respectively, *p < 0.05*). These data indicate that the reactions leading to the formation of the yellow colour were increasingly activated by the increase in temperature up to 99.8°C.

The reduction in the total carbohydrate of fresh coconut water during storage (Figure 6) was the result of reactions that use sugars and other simple carbohydrates in the formation of the insoluble matter, (the base substance of the coconut endosperm) which resulted in the increase in turbidity (Figure 1). Autoclaving, with its associated high temperature and pressure, is known to reduce nutritional value of media through the activation of a myriad of reaction involving amino acids, sugars, proteins and other carbohydrates [27,28]. This, most likely resulted in the reduction of its total carbohydrate (Figure 6). Gamma irradiation leads to the formation of reactive radicals with little potential of being involved in reactions such as those associated with sugars, as such, a marginal reduction in total carbohydrate was observed for Irradiated coconut water.

Considering the changes observed thus far, it would be convenient to measure the increase in bacteria cells (growth) by measuring the dry biomass or turbidity of bacteria culture in fresh, autoclaved or gamma irradiated coconut water with out storage. This is because prior to storage, all of the three coconut water samples recorded low turbidity, low dry matter (< 1.5 mg/mL) and a wavelength of maximum absorbance of less than 600 nm. These would not obscure small changes in absorbance/turbidity of a growing bacteria culture, measured at wavelengths equal to or greater than 600 nm. However, for both stored fresh and gamma irradiated coconut water, the high and increasing turbidity and dry matter content (> 1.5 mg/mL) during storage imply possible difficulties in recording small changes during the measurements of turbidity or dry biomass (particularly at the lag phase) of a growing bacteria culture. The same possibility of inconsistencies and non-reproducibility is expected with autoclaved coconut water because of the decrease and subsequent increase in its turbidity and dry matter despite that these were low.

## Conclusion

Sterilization of fresh coconut water by either autoclaving or low dose gamma irradiation led to the development of an intense or pale yellow colour respectively. Also associated with these, were different extents of variations in turbidity, dry matter and pH. The storage of fresh coconut water at 4°C for both two and four weeks resulted in the development of an intense yellow colour and a striking change in turbidity, dry matter and pH.

The use of turbidity measurement for measuring the growth of bacteria in fresh, autoclaved and gamma irradiated coconut water is practicable without any possibility of interference by the very low turbidity and bright colours of these different coconut waters. However, this may result in consistencies and, or non-reproducible data if stored fresh, stored autoclaved or stored gamma irradiated coconut water were used.

## Abbreviations

FCW: Fresh coconut water; RCW: Irradiated coconut water; ACW: Autoclaved coconut water; SFCW: Stored fresh coconut water; SRCW: Stored irradiated coconut water; SACW: Stored autoclaved coconut water.

## Competing interests

The authors declare that they have no competing interests.

## Authors' contributions

AKA conceived the study, and was involved with its design and the performance of all laboratory analyses, statistical analyses and drafted the manuscript. EDD participated in the design of the study and performance of all laboratory analyses, RA was involved with the design, acquisition of samples and participated in the performance of all the laboratory analyses and the drafting of the manuscript. All authors read and approved the final manuscript.
